# Prevalence and Outcomes of Orthostatic Hypotension in Hemorrhagic Stroke Patients During Hospitalization

**DOI:** 10.3390/neurolint16060134

**Published:** 2024-12-20

**Authors:** Pui Kit Tam, Guhan Ramamurthy, Lavanya Rawat, Serene Huang, Jeong Hoon Lim

**Affiliations:** 1 Division of Rehabilitation Medicine, Department of Medicine, National University Hospital, Singapore 119228, Singapore; mdctpk@nus.edu.sg (P.K.T.);; 2 Department of Medicine, Yong Loo Lin School of Medicine, National University of Singapore, Singapore 117549, Singapore; 3 BG Institute of Neurosciences, BG Hospital, Tiruchendur, Tuticorin 628216, Tamil Nadu, India

**Keywords:** stroke, hemorrhage, orthostatic hypotension, surgery

## Abstract

Background/Objectives: Orthostatic hypotension (OH) is highly prevalent in hospitalized patients and can lead to major consequences. The prevalence of OH among patients with stroke has also been reported to be high in in-patient cohorts. However, no previous analysis has focused exclusively on patients with hemorrhagic stroke, a group that may have a different disease profile, including a greater need for blood pressure control and surgical intervention. This study aims to examine the prevalence of OH, its risk factors, and potential impact in patients who were hospitalized due to hemorrhagic stroke. Methods: A retrospective analysis of in-patient records between 1 January 2021 and 30 April 2023 was conducted for patients with stroke due to intracerebral hemorrhage (ICH) or subarachnoid hemorrhage (SAH) who were referred to rehabilitation at a tertiary hospital in Singapore. OH was defined as a drop in systolic blood pressure of ≥20 mmHg or diastolic blood pressure of ≥10 mmHg during the sit-up test as part of the rehabilitation assessment. Additional data collected included demographic information, length of stay, antihypertensive medications used at the time of assessment, comorbidities, and discharge functional outcomes as measured by a modified Rankin Scale. Results: A total of 77 patients (65 [84.4%] with ICH and 12 [15.6%] with SAH) were included in the analysis. The prevalence of OH was 37.7%. A history of surgical intervention was identified as the major risk factor for the development of OH (odds ratio 4.28, 95% confidence interval 1.37 to 13.35, *p* = 0.009). There was no difference in hospital length of stay or discharge modified Rankin Scale scores between the two groups. Conclusions: OH was frequently observed among patients with hemorrhagic stroke during the acute/subacute stage and should be monitored, especially in patients who require surgical intervention.

## 1. Introduction

Orthostatic hypotension (OH), defined as a 20 mmHg or 10 mmHg drop in systolic or diastolic blood pressure (BP) respectively, when the patient assumes an upright position from a supine position within 3 min [1], results from a decreased venous return on assuming an upright position due to the pooling of blood in the peripheries and splanchnic circulation owing to the defective compensatory increase in the vascular tone that is needed to maintain the patient’s blood pressure. It is highly prevalent in hospitalized patients occurring in as many as 75% in the elderly population [2] with potentially severe consequences including falls and syncope, and, in the longer term, ischemic stroke, major cardiovascular events, and all-cause mortality [3]. OH occurring in patients undergoing acute rehabilitation is associated with a prolonged length of stay (LOS) and reduced rehabilitation efficacy, regardless of the admission diagnosis [4].

Stroke is one of the top causes of hospitalizations [5]. The reported prevalence of OH among patients with stroke varies significantly, because of substantial differences in the timing of the measurement, the etiology of stroke included, and the method of detection. In patients during the first week of ischemic stroke with mild to moderate severity, the incidence of OH was reported to be around 10% [6]. It was reported that OH was present in 52% of stroke patients undergoing subacute stage rehabilitation [7]. In longitudinal studies of patients with stroke and transient ischemic attacks in the community, the prevalence of OH was up to 39% [3]. Regarding the consequences of OH specifically in stroke patients, a transcranial Doppler ultrasound study on patients with ischemic stroke of the middle cerebral artery territory showed reduced blood flow velocity in the affected side of the brain [8] and increased risk of recurrent stroke in the long run [3].

Apart from one case report [9], to date there has been no fully committed study regarding OH in patients with hemorrhagic stroke to describe its clinical features, identify possible contributing factors, and gauge clinical impact on the outcome. Previous studies involving both ischemic and hemorrhagic strokes found no differences in OH prevalence between the two types, and OH did not impact length of stay, functional outcomes, cardiovascular risks, or mortality [7,10]. However, hemorrhagic stroke data in these studies were limited. Lowering blood pressure is recommended in acute intracerebral hemorrhage [11]. Additionally, OH is common after major surgeries, contributing to delayed mobilization [12,13], falls, and prolonged hospital stay [14,15]. Therefore, OH in hemorrhagic stroke patients should be studied separately from ischemic stroke.

This study aims to examine the prevalence of OH in patients with hemorrhagic stroke, identify potential risk factors, and evaluate its clinical impact. A retrospective analysis was conducted on cases of hemorrhagic stroke referred for rehabilitation assessment at a tertiary hospital in Singapore. The findings will help clinicians identify high-risk patients earlier, mitigate the adverse effects of OH, and provide preliminary data prior to conducting a prospective study.

## 2. Materials and Methods

### 2.1. Patient Selection

This was a retrospective analysis of consecutive patients referred to rehabilitation medicine between 1 January 2021 and 30 April 2023 at National University Hospital, Singapore. We included all patients aged 18–99, with a diagnosis of “stroke due to intracerebral hemorrhage” (ICH) or “stroke due to subarachnoid hemorrhage” (SAH) according to the American Heart Association/American Stroke Association diagnostic criteria [16]. Exclusion criteria included the absence of postural blood pressure measurements during their hospitalization, patients with hemorrhages due to trauma, tumors, or the hemorrhagic transformation of an ischemic infarct.

### 2.2. Postural BP Measurement

As part of the rehabilitation assessment during hospitalization for stroke patients, BP was measured by the nursing team using the sit-up test [17]. BP was first measured while the patient rested in a supine position, followed by BP measurement after the patient sat erect for 3 min. OH was defined as a drop in systolic BP of ≥20 mmHg or diastolic BP of ≥10 mmHg when compared to the supine BP. The date when OH was first detected was recorded. For patients without OH throughout hospitalization, the first date of postural BP measurement was used. We analyzed and compared the groups with at least one reading meeting the OH criteria during the hospital stay to those without any documented instances of OH.

### 2.3. Additional Data Collection

Additional information collected included any surgical interventions performed for the stroke, demographic data (age, gender), diagnosis (ICH or SAH), length of stay in the intensive care unit (ICU)/high-dependency unit (HDU), acute general ward, and rehabilitation ward. The use of antihypertensive medications was identified as an important risk factor for OH [18]. We documented the administration of these medications within 48 h before the postural blood pressure assessment by reviewing patients’ prescription records. Co-morbidities known to be associated with an increased risk of OH were also recorded, including hypertension, diabetes, chronic kidney disease, ischemic heart disease, prior stroke, and Parkinsonism [18]. Functional outcomes at hospital discharge were evaluated using the modified Rankin Scale (mRS). To assess severity, hematoma volume for ICH patients was calculated using the ABC/2 formula [19], while the World Federation of Neurological Surgeons (WFNS) grading scale [20] was documented for SAH patients.

### 2.4. Statistical Analysis

Statistical analysis was performed using IBM SPSS version 27. The Shapiro–Wilk test was used to test for normality in continuous variables. Normally distributed variables were expressed as mean (standard deviation, SD), independent *t*-tests and the paired *t*-test were used for between-group and within-group comparisons, respectively. Non-normally distributed variables and ordinal scales (mRS and WFNS grading scale) were expressed as median (interquartile range, IQR), and Mann–Whitney U tests were used for comparisons between groups. Chi-square tests were used for categorical variables. Statistical significance was set at 0.05.

### 2.5. Ethical Approval

The study was approved by the National Healthcare Group Domain-Specific Review Board of Singapore (2023/00489). A waiver of consent was obtained as the study involved only collecting non-sensitive data during routine clinical care, with minimal to no risk to patients. Measures to ensure patient confidentiality were strictly implemented.

## 3. Results

**Figure 1:**
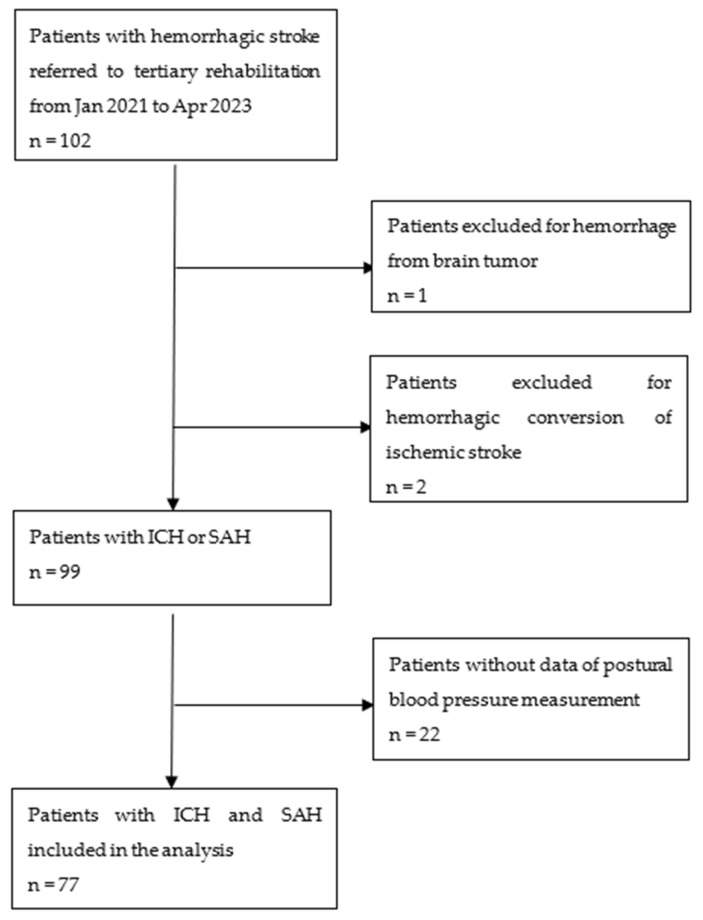
Case selection process. Abbreviations: ICH, intracranial hemorrhage; SAH, subarachnoid hemorrhage.

**Figure 2:**
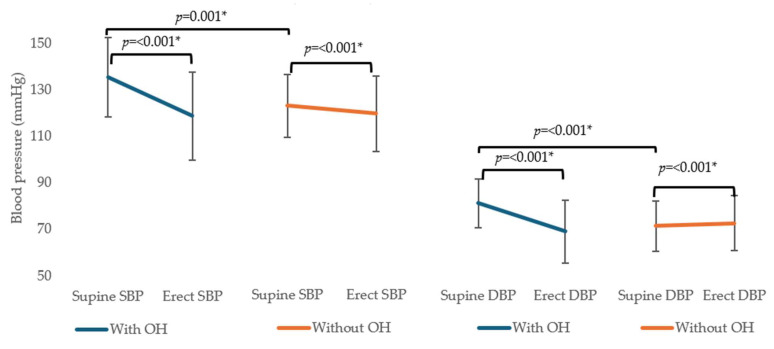
Postural blood pressure measurement. Abbreviations: OH, orthostatic hypotension; SBP, systolic blood pressure; DBP, diastolic blood pressure. * *p* < 0.05, statistically significant.

A total of 102 cases with hemorrhagic stroke were identified during the study period. Three cases were excluded due to the presence of tumors or hemorrhagic transformation of ischemic stroke. Twenty-two cases were excluded due to the unavailability of postural BP records. Ultimately, 77 cases were included in the analysis (Figure 1). The mean age was 58.4 years (SD 12.0), with 65 (84.4%) ICH and 12 (15.6%) SAH cases. The OH measurement was conducted approximately in the second week after the stroke [12.0 (IQR 14.0) days]. Twenty-nine (37.7%) cases had OH, with twenty patients (26.0%) meeting the diastolic BP criteria, sixteen (20.8%) meeting the systolic BP criteria, and seven (9.1%) meeting both criteria. Supine BP measurements were significantly higher (both systolic and diastolic) in the group with OH than the group without OH. The group with OH had both systolic and diastolic BP drop significantly after 3 min in the erect position, while the group without OH had systolic BP drop, but slight elevation of diastolic BP in the erect position (Figure 2).

The groups with and without OH had similar ages, lengths of stay in the ICU/HDU, overall lengths of hospital stay, and numbers of antihypertensive medications. The prevalence of co-morbidities was also similar between the two groups. None of the subjects suffered from Parkinsonism. However, significantly more patients in the group with OH had undergone surgical interventions during admission (37.9% vs. 12.2%, *p* = 0.008) (Table 1). The most commonly performed operation was insertion of extra-ventricular drain but overall, no particular type of surgical intervention was found to be more related to the development of OH (Supplementary Table S1).

We also analyzed the data by type of hemorrhage. The severity of hemorrhage, as measured by ICH hematoma volume and the WFNS grading scale, did not significantly differ between patients with and without OH (Supplementary Table S2). The prevalence of OH among ICH and SAH patients was 35.4% and 50%, respectively.

Due to a change in the hospital’s electronic record system, we were unable to retrieve medication lists for 12 patients (15.6%). Among those with available medication lists, both groups received a similar number of antihypertensive medications [2.5 (IQR 2.5) vs. 2.0 (IQR 1.0), *p* = 0.721]. No specific class of antihypertensive medication was found to be more strongly associated with OH.

The odds ratio for developing OH in patients who had surgical interventions compared to those without was 4.28 (95% confidence interval 1.37 to 13.35, *p* = 0.009).

## 4. Discussion

To the best of our knowledge, this is the first analysis of orthostatic hypotension among patients with hemorrhagic stroke. We found that patients who required surgical intervention for a brain hemorrhage were the most susceptible to the development of OH.

The mechanism for the development of OH after stroke is likely multifactorial. Autonomic dysregulation [21] and muscle weakness caused by stroke may impair the normal physiological mechanisms that counteract BP drops with postural changes. Co-morbidities such as diabetes and heart failure are also important risk factors for OH [22]. Arterial stiffness, common in stroke patients, can lead to a blunted orthostatic increase in arterial pressure [23]. The use of medications, including antihypertensives and diuretics, may also contribute to OH. In addition, prolonged immobility can result in cardiovascular deconditioning, muscle atrophy, increased venous pooling, and hypovolemia.

Diastolic OH was more frequent than systolic OH, which differed from studies of OH in the community-dwelling population, where systolic OH was typically 2–3 times more common than diastolic OH [24,25]. However, this finding was similar to reports from in-patient populations [2,26]. In fact, the orthostatic response in healthy subjects typically shows a 10–15% increase in diastolic BP, with the systolic BP remaining relatively unchanged [27]. The orthostatic response in those without OH more closely resembled that of healthy subjects (Figure 2). Prolonged bed rest may impair baroreceptor-mediated sympathetic vasomotor activity [28]. Significant hypovolemia can cause a drop in diastolic BP during the orthostatic response [29]. As systemic vascular resistance, circulatory volume, and skeletal muscle pumping action are important determinants of the diastolic response to orthostatic stress [22], these factors may explain why hospitalized patients, particularly those with stroke, are more likely to have diastolic OH than community-dwelling individuals. In a similar context, patients who require surgical interventions for hemorrhage are often immobilized for longer periods, which could contribute to a higher prevalence of OH in this group. Although we could not find any reports specifically on the prevalence of OH in patients who required surgery for hemorrhagic stroke, reduced heart rate variability and OH have been reported after other types of surgeries, potentially related to opioid use and postoperative inflammatory responses [12,30]. Previous studies also noted that patients with OH had higher supine systolic and diastolic BP than those without OH [10,26]. Supine hypertension is commonly seen in patients with OH and autonomic dysfunction [22] may explain this phenomenon.

The prevalence of OH in our study was lower than in Kong’s study in a similar rehabilitation setting in Singapore for patients with stroke [7]. This could be due to the following reasons. Firstly, our study did not include patients with ischemic stroke and the prevalence of diabetes (18.2%) was lower than in Kong’s study (36.6%). Given that diabetes is a known factor for autonomic neuropathy and the proportion of patients having diabetes is lower in hemorrhagic than ischemic stroke [31], the prevalence of OH might be lower in our cohort. However, the length of stay and functional outcome for those with OH did not differ from those without, which is consistent with Kong’s findings and may imply the effect of appropriate clinical interventions to minimize the impact of OH during the rehabilitation stay.

Beta-blockers and alpha-blockers are thought to be more likely to precipitate OH than other classes of antihypertensives [32]. However, in our analysis, none of the drugs were found to be particularly more likely to cause OH. Similarly, age and the prevalence of co-morbidities, such as diabetes and heart disease, which are known risk factors, were not significantly different in the OH group. The relatively younger population and fewer co-morbidities compared with previous studies might explain this difference [26]. There are likely complex interactions between these risk factors and the development of OH. The small sample size and the retrospective nature of our study might limit the ability to detect the interplay of risk factors.

There are some technical limitations regarding the method used to measure OH in this study. The most widely used method to detect OH in research studies involves measuring BP after 1 and 3 min of standing [18,22]. However, stroke patients may have difficulty maintaining a standing posture, and tilt-table testing is not readily available in clinical settings. The sit-up test, which we used in our study, has also been employed in some studies involving stroke patients [33]. A recent study proposed that a smaller cutoff of a 10 mmHg drop in systolic BP or a 5 mmHg drop in diastolic BP may be more optimal when using the sit-up test to detect OH [34]. If we followed this new criterion, the prevalence in our cohort would increase to 61%. Additionally, the reproducibility of OH measurements is poor, and multiple measurements are often required to detect OH [35]. In the acute phase of stroke, sympathetic overactivity may make OH less common when measured in the first week compared to later phases [36,37]. In this study, postural BP measurements were taken around 2 weeks after the stroke. For those detected with OH, monitoring postural BP responses longitudinally would provide important insights for managing and predicting the prognosis of these patients. Another limitation is the lack of data on unified initial clinical severity, such as the National Institutes of Health Stroke Scale, which could be a confounding factor. Beyond the discharge-modified Rankin Scale, we did not have other outcome measures such as falls, readmissions, or functional outcomes after discharge from the hospital. Due to the retrospective nature of the study, there were also a significant number of subjects having no postural BP measurements. This may potentially lead to bias because patients who were at higher risk or were more symptomatic might be more likely to have their postural BP measured and thus the prevalence in our cohort might be overestimated. Lastly, the missing medication data of the 12 patients might affect the interpretation of the effects of antihypertensives on OH.

## 5. Conclusions

Based on this preliminary retrospective study, the prevalence of OH during in-patient stay for patients with hemorrhagic stroke was estimated to be 37.7%. A history of surgical intervention for stroke was the most significant risk factor, and OH based on diastolic BP criteria was more commonly observed than OH based on systolic criteria. However, there were no differences in the overall length of stay or functional outcomes between patients with and without OH. Given the limitations of this retrospective study, well-designed prospective research would be warranted to investigate the true prevalence of OH and its clinical implications in patients with hemorrhagic stroke, and the mechanism of OH in those who had surgical intervention.

## Figures and Tables

**Table 1:** Characteristics of patients with and without orthostatic hypotension.

		With OH *n* = 29 ^#^	Without OH *n* = 48 ^#^	*p* Value
Age, mean (SD)		60.1 (13.0)	57.4 (11.3)	0.338
Gender, *n* (%)	Male Female	18 (62.1%) 11 (37.9%)	31 (64.6%) 17 (35.4%)	0.824
Type of stroke, *n* (%)	ICH	23 (79.3%)	42 (87.5%)	0.351
	SAH	6 (20.7%)	6 (12.5%)	
Time from onset to when OH test was completed (days), median (IQR)		11.0 (11.0)	13.5 (15.0)	0.218
Surgical intervention performed *n* (%)		11 (37.9%)	6 (12.5%)	0.009 *
Length of stay (days), median (IQR)	ICU/HDU	5.0 (6.0)	3.0 (3.0)	0.507
	Acute general ward	10.0 (9.0)	7.0 (9.8)	0.087
	Rehabilitation ward	28.0 (15.0)	29.0 (21.0)	0.724
	Total	45.0 (27.0)	41.0 (31.0)	0.535
Co-morbidities, *n* (%)	Prior stroke	6 (20.7%)	8 (16.7%)	0.657
	Diabetes	5 (17.2%)	9 (18.8%)	0.868
	Chronic kidney disease	2 (6.9%)	0 (0.0%)	0.139
	Ischemic heart disease	3 (10.3%)	4 (8.3%)	0.766
	Hypertension	22 (75.9%)	33 (68.8%)	0.503
Antihypertensives^#^	ACEI or ARB	18 (75.0%)	28 (68.3%)	0.566
	Alpha-blocker	4 (16.7%)	6 (14.6%)	0.827
	Beta-blocker	11 (45.8%)	24 (58.5%)	0.321
	Calcium channel blocker	20 (83.3%)	34 (82.9%)	0.966
	Diuretics	2 (8.3%)	0 (0.0%)	0.133
	Hydralazine	8 (33.3%)	8 (19.5%)	0.212
Total number of antihypertensives ^#^, median (IQR)		2.5 (2.5)	2.0 (1.0)	0.721
mRS on discharge, median (IQR)		4.0 (2.0)	4.0 (2.0)	0.548

^#^ There was missing data for antihypertensive information (with OH, *n* = 23; without OH, *n* = 42). * *p* < 0.05, statistically significant. Abbreviations: OH, orthostatic hypotension; SD, standard deviation; IQR, interquartile range; BP, blood pressure; ICH, intracerebral hemorrhage; SAH, subarachnoid hemorrhage; ACEI, angiotensin-converting enzyme inhibitor; ARB, angiotensin receptor blocker; mRS, modified Rankin Scale; ICU/HDU, intensive care unit/high-dependency unit.

## Data Availability

The data presented in this study are available on request from the corresponding author.

## Supplementary Materials

The following supporting information can be downloaded at: https://www.mdpi.com/article/10.3390/neurolint16060134/s1, Table S1: Type of surgical interventions performed; Table S2: Subgroup analysis according to type of hemorrhage.
