# Supplementary material for: Prevalence and Outcomes of Orthostatic Hypotension in Hemorrhagic Stroke Patients During Hospitalization
Source: Neurol Int. 2024 Dec 20;16(6):1878–86. doi: 10.3390/neurolint16060134 (PMC11678119; doi:10.3390/neurolint16060134)
Supplement: Supplementary file 1 [file neurolint-16-00134-s001.zip › neurolint-3373109-supplementary.pdf]

Supplementary Table S1. Type of surgical interventions performed.

Type of surgical intervention	With orthostatic hypotension *n* = 11	Without orthostatic hypotension *n* = 6	*p* Value
Extra-ventricular drain insertion	9 (81.8%)	5 (83.3%)	0.938
Clot evacuation	4 (16.7%)	1 (36.4%)	0.600
Craniectomy	4 (36.4%)	1 (16.7%)	0.600
Insertion of intracranial pressure monitor	4 (36.4%)	1(16.7%)	0.600
Clipping of aneurysm	3 (27.3%)	0 (0.0%)	0.515

Data expressed as n (%).

Supplementary Table S2. Subgroup analysis according to type of hemorrhage.

a. Intracerebral hemorrhage

		With OH *n* = 23[#]	Without OH *n* = 42[#]	*p* Value
Age, mean (SD)		60.7 (14.1)	57.7 (11.5)	0.360
Gender, *n* (%)	Male	15 (65.2%)	27 (64.3%)	0.940
	Female	8 (34.8%)	15 (35.7%)	
Time from onset when OH test done (days), median (IQR)		9.0 (7.0)	12.5 (14.0)	0.155
Surgical intervention done, *n* (%)		5 (21.7%)	2 (4.8%)	0.087
Total hospital length of stay (days), median (IQR)		39.0 (23.0)	37.5 (26.0)	0.399
Co-morbidities, *n* (%)	Prior stroke	5 (21.7%)	8 (19.0%)	0.795
	Diabetes	4 (17.4%)	9 (21.4%)	0.758
	Chronic kidney disease	2 (8.7%)	0 (0.0%)	0.122
	Ischemic heart disease	3 (13.0%)	4 (9.5%)	0.691
	Hypertension	18 (78.3%)	30 (71.4%)	0.549
Antihypertensives[#]	ACEI or ARB	16 (76.2%)	26 (74.3%)	0.873
	Alpha-blocker	4 (19.0%)	6 (17.1%)	0.857
	Beta-blocker	11 (52.4%)	21 (60.0%)	0.577
	Calcium channel blocker	19 (90.5%)	31 (88.6%)	0.823
	Diuretics	2 (9.5%)	0 (0.0%)	0.136
	Hydralazine	8 (38.1%)	8 (22.9%)	0.222
Total number of antihypertensives[#], median (IQR)		3.0 (2.0)	2.0 (1.0)	0.569
mRS on discharge, median (IQR)		4.0 (2.0)	4.0 (2.0)	0.693
ICH volume (ml), mean (SD)		15.8 (15.9)	10.9 (12.2)	0.173

b. Subarachnoid hemorrhage

		With OH *n* = 6[α]	Without OH *n* = 6[α]	*p* Value
Age, mean (SD)		58.0 (7.8)	55.3 (11.3)	0.645
Gender, *n* (%)	Male	3 (50.0%)	4 (66.7%)	0.558
	Female	3 (50.0%)	2 (33.3%)	
Time from onset when OH test done (days), median (IQR)		15.5 (19.0)	21.0 (22.0)	0.631
Surgical intervention done, *n* (%)		6 (100.0%)	4 (66.7%)	0.455
Total hospital length of stay (days), median (IQR)		52.5 (35.0)	61.5 (53.0)	0.423
Co-morbidities, *n* (%)	Prior stroke	1 (16.7%)	0 (0.0%)	0.500
	Diabetes	1 (16.7%)	0 (0.0%)	0.500
	Chronic kidney disease	0 (0.0%)	0 (0.0%)	1.000
	Ischemic heart disease	0 (0.0%)	0 (0.0%)	1.000
	Hypertension	4 (66.7%)	3 (50.0%)	1.000
Antihypertensives[α]	ACEI or ARB	2 (66.7%)	2 (33.3%)	0.524
	Alpha-blocker	0 (0.0%)	0 (0.0%)	1.000

	Beta-blocker	0 (0.0%)	3 (50.0%)	0.464
	Calcium channel blocker	1 (33.3%)	3 (50.0%)	0.595
	Diuretics	0 (0.0%)	0 (0.0%)	1.000
	Hydralazine	0 (0.0%)	0 (0.0%)	1.000
Total number of antihypertensives[α], median (IQR)		1.0 (1.0)	1.0 (3.0)	0.788
mRS on discharge, median (IQR)		4.0 (1.25)	4.0 (3.0)	0.604
WFNS grading scale, median (IQR)		2.0 (2.25)	4.0 (3.5)	0.703

[#]There was missing data for the information regarding antihypertensives (with OH, $n = 21$; without OH, $n = 35$). [α]There was missing data for the information regarding antihypertensives (with OH, $n = 3$; without OH, n=6). *$p < 0.05$ statistically significant. Abbreviations: OH, orthostatic hypotension; SD, standard deviation; IQR, interquartile range; BP, blood pressure; ACEI, angiotensin converting enzyme inhibitor; ARB, angiotensin receptor blocker; mRS, modified Rankin Scale; ICH, intracerebral hemorrhage; WFNS, World Federation of Neurological Surgeons.
